# Abortion

**Published:** 1883-07

**Authors:** A. S. Heath

**Affiliations:** Professor of Bovine,, Ovine and Suvine Pathology, Columbia Veterinary College


### ART. XX.—ABORTION.

BY A. S. HEATH, M.D.

*Professor of Bovine,, Ovine and Suvine Pathology, Columbia Veterinary College.*

MISCARRIAGE, slinking, abortion, or slipping the calf, occurs to cows in apparent high health and condition. It is usual to divide the period of gestation of forty-two weeks or two hundred and eighty days, into two parts; the first part, six months; second, three months. To all cases occurring during the first part the term abortion is applied, and after that premature labor.

*Causes.*—These may be divided into external, internal, predisposing and exciting.

The external causes are those due to atmospherical influences, bad sanitary conditions and to the various accidents in field, pasture, road or stable.

The internal causes are dependent upon disease, malformation, or malposition of the young in utero.

The predisposing causes may be previous abortion, hereditary predisposition, uterine disease, either by extension or sympathy, disease conveyed from mother to fœtus, pleuro-pneumonia, great size of fœtus, or more than one; extreme plethora or obesity of the mother, often become predisposing causes.

Exciting causes, are sudden and extreme changes of temperature, bad, or indigestible food, smut rye grass, diseased or mouldy corn, food too stimulating, excessive drinking of cold water, foul water, eating horse-tail grasses, sedges, hellebore, savin, rue, ergot of rye, the external or internal use of cantharides, turpentine, or drastic purgatives.

*Treatment.*—Change of pasture and attention to sanitary regulations, enemas of warm water, laxative, e. g.

| | |
|---|---|
| ℞ Epsom salts, | 12 oz. |
| Sulphur, | 3 " |
| Ginger and Gentian, ã ã | 1 " |

Give in molasses and water, one-quarter to sheep.

When the uterus lacks power to expel the fœtus, and when abortion cannot be prevented, give

℞ Aromat. spts. ammonia, 2 to 3 oz.
Tr. cardamons, 1 "

Give in a quart of ale one-quarter to sheep, or,

℞ Plv. ergot, 4 drs.
Ginger, plv., 1 oz.

Give in a pint of ale every hour.

The placenta and membranes, if long retained and offensive, should be removed by the hand.

℞ Solut. carbolic acid, glycerine, ã ã ½ oz.

Water, ½ gal.

Inject into vagina,

*Premature Labor.*—The longer the fœtus remains in place the better prepared is nature for delivery.

Malposition or death of fœtus may cause the premature labor. The animal suffers with unusual pains. If a foot protrudes and the hair comes off readily, the calf is dead. Debility of the female indicates danger and demands artificial delivery and stimulants.

*To Prevent Abortion.*—Feed a pint of hemp seed daily for seven months.

Feed a pint of flax-seed to cows ready to "come in," and for three or four days after.

Suckle the calves twice a day if not fed. It is advisable to let the calves suck the cows for at least a week, as it is a return to nature.

